# The Role of Charge Balance and Excited State Levels on Device Performance of Exciplex-based Phosphorescent Organic Light Emitting Diodes

**DOI:** 10.1038/s41598-017-12059-2

**Published:** 2017-09-20

**Authors:** Sangyeob Lee, Hyun Koo, Ohyun Kwon, Young Jae Park, Hyeonho Choi, Kwan Lee, Byungmin Ahn, Young Min Park

**Affiliations:** 1 Samsung Advanced Institute of Technology, Samsung Electronics Co., Ltd, 130 Samsung-ro, Yeongtong-gu, Suwon, Gyeonggi 16678 Korea; 2 Department of Materials Science and Engineering and Energy Systems Research, Ajou University, 206 Worldcup-ro, Yeongtong-gu, Suwon, Gyeonggi 16499 Korea; 3 Surface Technology Group, Korea Institute of Industrial Technology (KITECH), Incheon, 21999 Republic of Korea

## Abstract

The design of novel exciplex-forming co-host materials provides new opportunities to achieve high device performance of organic light emitting diodes (OLEDs), including high efficiency, low driving voltage and low efficiency roll-off. Here, we report a comprehensive study of exciplex-forming co-host system in OLEDs including the change of co-host materials, mixing composition of exciplex in the device to improve the performance. We investigate various exciplex systems using 5-(3–4,6-diphenyl-1,3,5-triazin-2-yl)phenyl-3,9-diphenyl-9H-carbazole, 5-(3–4,6-diphenyl-1,3,5-triazin-2-yl)phenyl)-9-phenyl-9H-3,9′-bicarbazole, and 2-(3-(6,9-diphenyl-9H-carbazol-4-yl)phenyl)-4-phenylbenzo[4,5]thieno[3,2-d]pyrimidine, as electron transporting (ET: electron acceptor) hosts and 9,9′-dipenyl-9H, 9′H-3,3′-bicarbazole and 9-([1,1′-biphenyl]-4-yl)-9′-phenyl-9H,9′H-3,3′-bicarbazole as hole transporting (HT: electron donor) hosts. As a result, a very high current efficiency of 105.1 cd/A at 10^3^ cd/m^2^ and an extremely long device lifetime of 739 hrs (t_95_: time after 5% decrease of luminance) are achieved which is one of the best performance in OLEDs. Systematic approach, controlling mixing ratio of HT to ET host materials is suggested to select the component of two host system using energy band matching and charge balance optimization method. Furthermore, our analysis on exciton stability also reveal that lifetime of OLEDs have close relationship with two parameters; singlet energy level difference of HT and ET host and difference of singlet and triplet energy level in exciplex.

## Introduction

Organic light-emitting diodes (OLEDs) have been rapidly developed since the first demonstration of multi-layered thin film structure by Tang *et al*.^1^. Especially, after discovery of the highly stable and efficient green light emitter tris(2-phenylpyridine)iridium(III), the OLED device efficiency has been significantly improved^2^. Due to these rapid developments, OLEDs have been commercialized as one of the next generation flat panel displays^3,4^. Yet, to broaden the market share of OLEDs in display panels, the enhancement in device electroluminescence (EL) efficiency and improved device lifetime are still required.

Device efficiency of OLED can be determined by external quantum efficiency (*η*_*ext*_)^5^:

1 $${\eta }_{ext}={\rm{\gamma }}\times {\eta }_{r}\times {\Phi }_{p}\times {\eta }_{p}$$

where *γ, η*_*r*_ *, Φ*_*p*_ *, η*_*p*_ are charge balance, exciton formation efficiency, quantum yield, device out-coupling efficiency, respectively. Various methods have been attempted to control these efficiency-controlling parameters of OLEDs and the efficiency of OLEDs has been improved significantly^5–8^_._ Recently, exciplex forming materials have attracted much attention as both emitting and host materials to fabricate an extremely high efficient OLED^9–12^. Exciplex shows red-shifted and broad photoluminescence (PL) spectra compared to its molecular (monomer) states. Thus, the emission properties of OLED can be tuned using exciplex-forming emitters and this concept was applied to white organic light-emitting diodes to achieve broad emission band^13,14^. The singlet-triplet gap of exciplex is small compared to its monomer state due to the small orbital overlap between the excited state and the ground state^15–17^. Goushi *et al*. demonstrated the OLED with enhanced device efficiency via. thermally activated delayed fluorescence of exciplex emitter due to the small exchange energy between singlet energy state (*S*_1_) and triplet energy state (*T*_1_)^18,19^. The small *S*_1_ − *T*_1_ gap can also be utilized to up-convert the triplet excitons to *S*_1_ in a host for the efficient energy transfer to a fluorescent dopant^20^. High-efficiency and low-driving voltage phosphorescent OLEDs using exciplex-forming co-hosts were also demonstrated for green^21^, blue^22–25^, orange^26^ and yellow^27,28^ emitting devices. These works attribute the high efficiency of devices to the low injection barrier of electron and hole to the exciplex-forming co-host and efficient energy transfer from exciplex in host to the guest dopant. Most of previous studies on the exciplex in OLEDs, however have been focused on the enhancement of device efficiency such as high EL efficiency, low operating voltage, and low efficiency roll-off mainly by engineering energy level for facile charge injection as well as controlling the doping level of dopants for efficient charge transfer. In this work, we investigate the parameters governing the improvement of device efficiency as well as device lifetime in OLEDs in the exciplex-forming co-host systems. In addition to the introduction of new high performance electron transporting (ET: electron acceptor) and hole transporting (HT: electron donor) host materials, we suggest that mixing ratio of ET to HT host materials in emission layer can be governing factors to allow for high device efficiency and long lifetime. In order to reveal the role of charge balance on device performance in the exciplex system, we compared three types of ET hosts which are expected to have different transport property. Our results clearly indicate that the mixing ratio of ET to HT host materials should be controlled as considering the transport properties of each layer to achieve charge balance. Furthermore, we found out optimal condition to enhance the device lifetime of exciplex-forming co-host system by considering excited state energy level difference between HT, ET molecules and exciplex molecules.

## Result and Discussion

**Figure 1:**
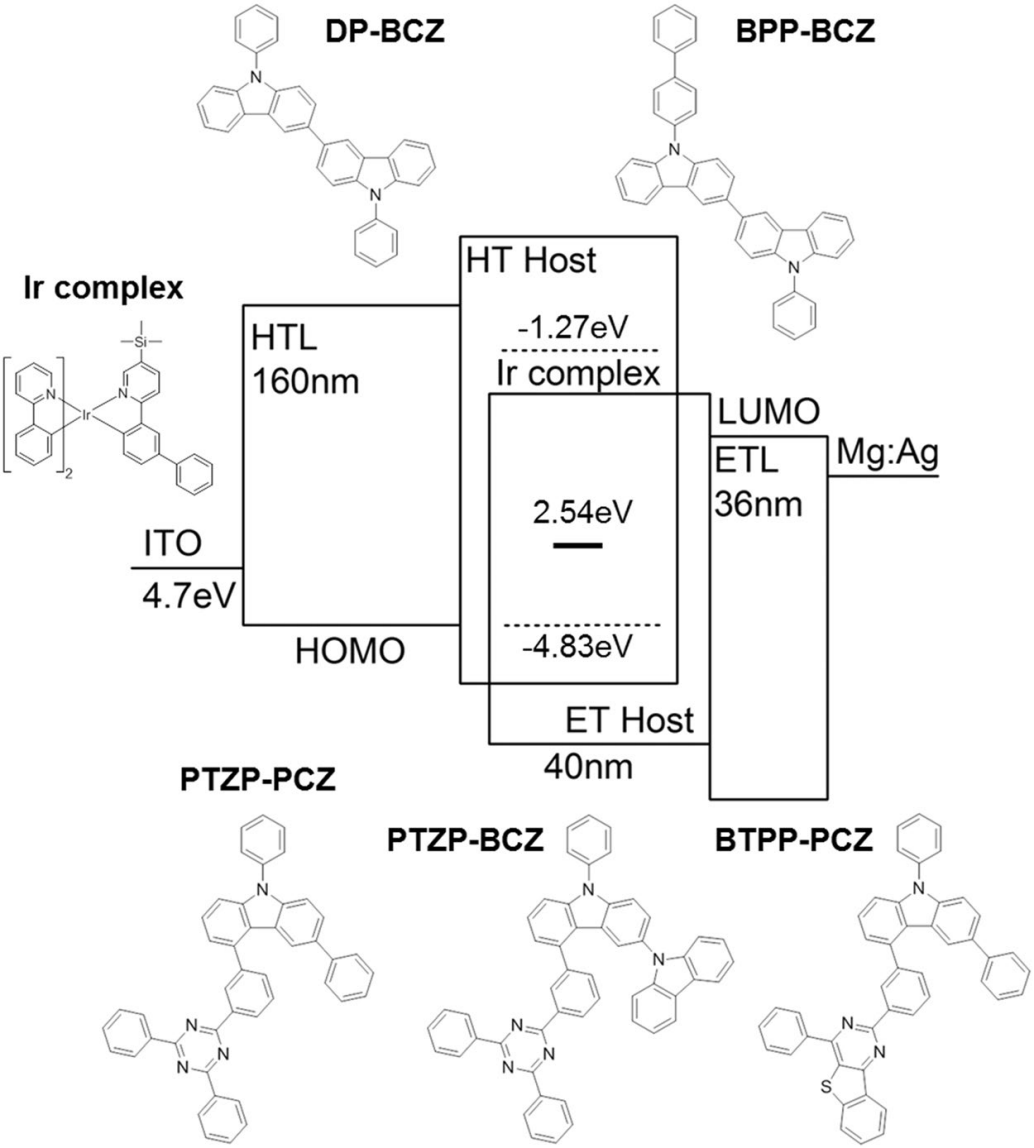
Proposed energy level diagrams of the mixed-host exciplex forming EML in top emission OLEDs. Relative HOMO and LUMO levels of HT host and ET host are shown schematically with respect to HOMO and LUMO of HTL and ETL. The chemical structure of HT hosts (DP-BCZ, BPP-BCZ), ET hosts (PTZP-PCZ, BTPP-PCZ, PTZP-BCZ) and Ir-complex dopant are indicated. The calculated value of T1 energy level of dopant, 2.54 eV is also indicated.

Figure 1 demonstrates a schematic diagram of the mixed-host exciplex forming top emission OLED systems and device structure fabricated in this work. As host materials, two types of HT {9,9′-dipenyl-9H, 9′H-3,3′-bicarbazole (DP-BCZ) and 9-([1,1′-biphenyl]-4-yl)-9′-phenyl-9H,9′H-3,3′-bicarbazole (BPP-BCZ)} and three types of ET {5-(3–4,6-diphenyl-1,3,5-triazin-2-yl)phenyl-3,9-diphenyl-9H-carbazole (PTZP-PCZ), 5-(3–4,6-diphenyl-1,3,5-triazin-2-yl)phenyl)-9-phenyl-9H-3,9′-bicarbazole (PTZP-BCZ), and 2-(3-(6,9-diphenyl-9H-carbazol-4-yl)phenyl)-4-phenylbenzo[4,5]thieno[3,2-d]pyrimidine (BTPP-PCZ)} molecules are employed, respectively, to compare exciplex formation and transport properties in the device. In order to form the emission layer, {2-([1,1′-biphenyl]-3-yl)-5-(trimethylsilyl)pyridinato-C^2^,*N*}-bis[2-phenylpyridinato-C^2^,*N*]iridium(III) [Ir(TMSBppy)ppy_2_], Ir-complex is doped via co-deposition. HT and ET hosts are paired to form exciplex in co-host systems having the highest occupied molecular orbital (HOMO) and the lowest unoccupied molecular orbital (LUMO) energy levels with differences larger than 0.07 eV and 1.0 eV, respectively. In addition, HT and ET hosts are specifically designed to have the HOMO and LUMO level difference with respect to HOMO and LUMO of hole transport layer (HTL) and electron transport layer (ETL) smaller than 0.3 eV and 0.15 eV, respectively to enhance hole/electron injection to emission layer (EML) (see Supplementary Table S1). Although energy levels of each host are designed to be well matched to the emission layer, the electron transport characteristic of ET molecules is expected to be stronger in an order of PTZP-PCZ, PTZP-BCZ, and BTPP-PCZ. Considering molecular structure of ET hosts, PTZP-PCZ and PTZP-BCZ are expected to exhibit high electron mobility resulting from stronger electron affinity due to the incorporation of triazine group compared to PTZP-PCZ having thieno-pyrimidine ET unit^29–31^. The existence of additional carbazole group in PTZP-BCZ that is commonly demonstrated to achieve ambipolar property of ET host, is also expected to reduce the electron transport property to some extent in spite of enhanced hole transport property^32,33^.

**Figure 2:**
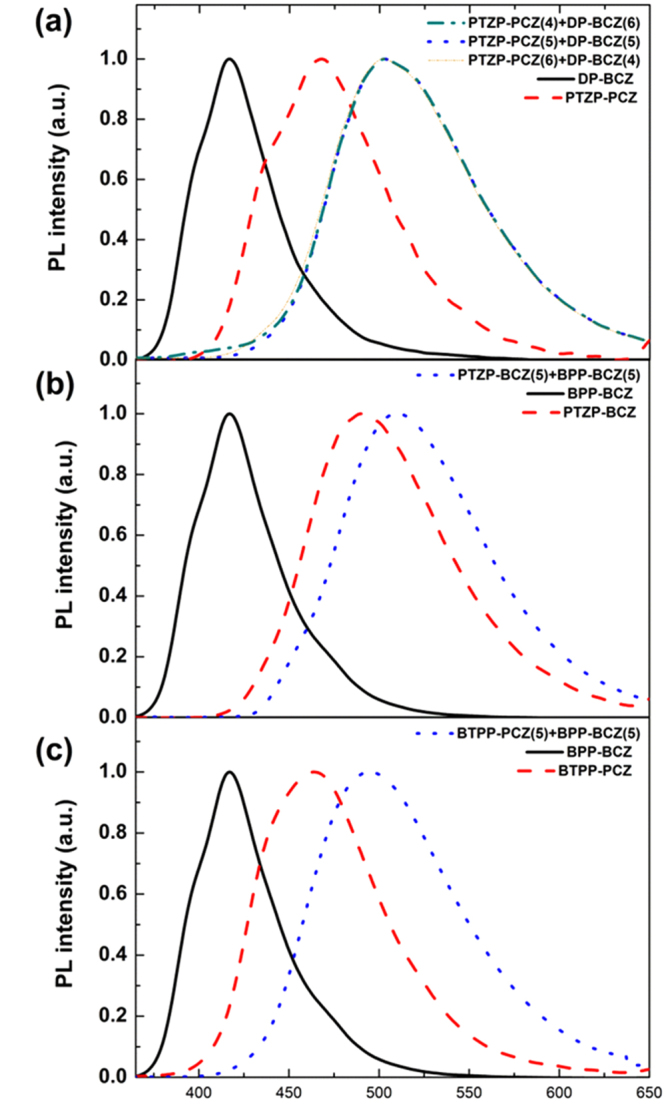
Normalized PL spectra of (**a**) PTZP-PCZ:DP-BCZ, (**b**) PTZP-BCZ:BPP-BCZ, and (**c**) BTPP-PCZ:BPP-BCZ co-deposited film. Note that PL spectra of PTZP-PCZ:DP-BCZ co-deposited film with mixing ration of 4:6 and 6:4 are indicated in Fig. 2(a).

To confirm the formation of exciplex, the photoluminescence (PL) spectra were taken in the three combinations, PTZP-PCZ:DP-BCZ, PTZP-BCZ:BPP-BCZ, and BTPP-PCZ:BPP-BCZ without dopant (Fig. 2). When mixed with a weight ratio of host, 1:1, the emission peaks were red-shifted without any trace of original emission peak of each HT and ET host, indicating that exciplex is well-formed in the mixed host upon excitation. The exciplex formed via co-deposition of HT and ET host showed emission peak around 2.407 eV close to the gap between the HOMO level of HT host and the LUMO level of HT host. It is notable that PL spectra of mixed host as increasing weight ratio of HT host to 4:6 [ET(PTZP-PCZ):HT(DP-BCZ)] also shows a similar emission trend to that of 5:5 weight ratio (Fig. 2a). This result suggests that slight change of weight ratio in ET and HT host does not make any significant effect on the uniform formation of exciplex in our co-host systems.

**Figure 3:**
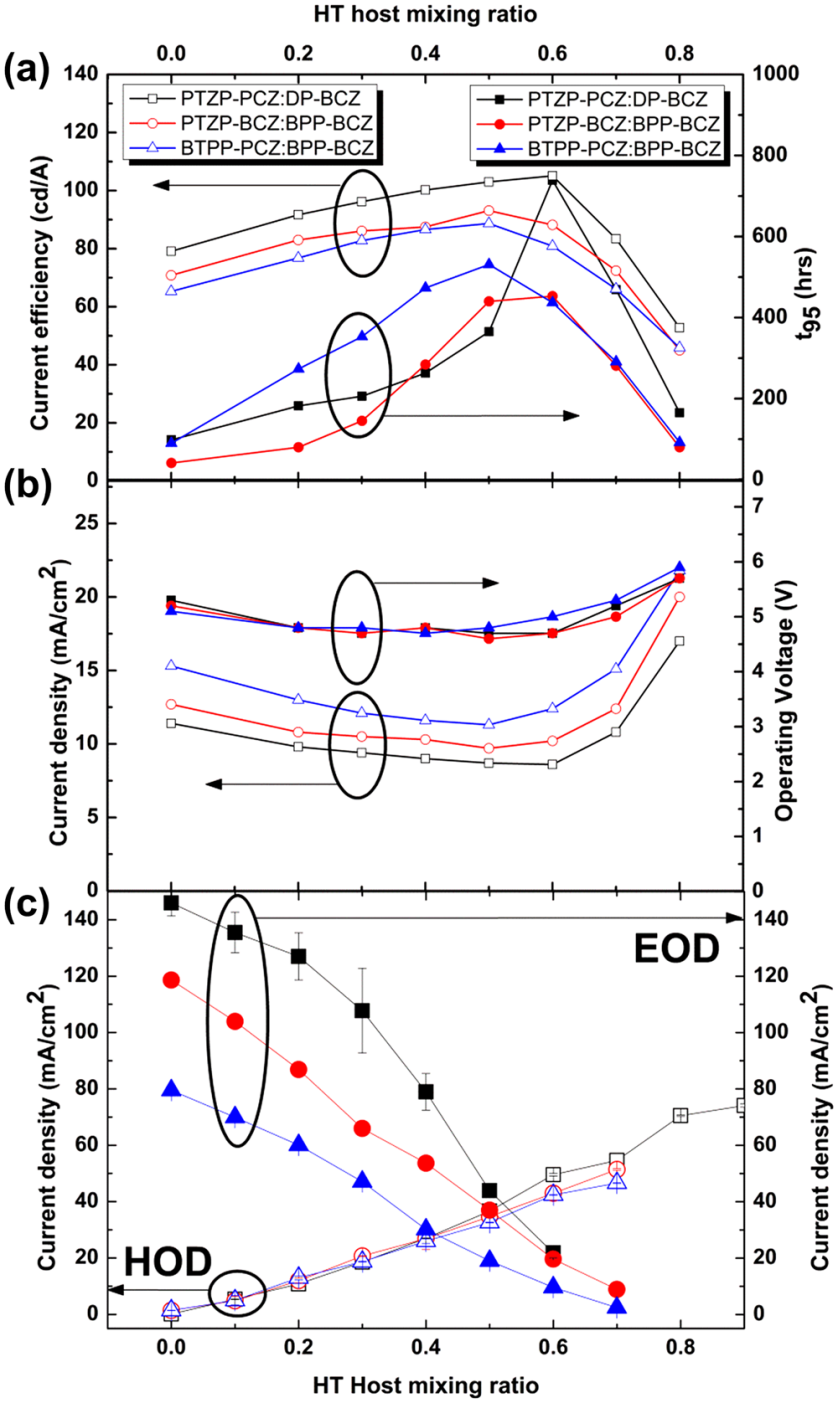
The effects of the EML mixing ratios on (**a**) the device efficiencies and lifetimes, (**b**) operating current densities and operating voltages of OLEDs with PTZP-PCZ:DP-BCZ, PTZP-BCZ:BPP-BCZ and BTPP-PCZ:BPP-BCZ two-host systems at luminance of 10^3^ cd/m^2^. (**c**) HOD and EOD of two host system with varying HT host mixing ratio.

Figure 3a,b compares the device characteristics with a variation of weight ratio of co-host, including current efficiencies, device lifetime, operating current density and operating voltage, at 10^3^ cd/m^2^ for three different two-host systems: PTZP-PCZ:DP-BCZ (set I), PTZP-BCZ:BPP-BCZ (set II) and BTPP-PCZ:BPP-BCZ (set III). Using HT and ET host materials separately as a mixed host system, charge balance can be controlled with an intended manner by controlling the composition of the two materials during deposition. The mixing ratio of HT hosts was varied from 0 to 0.8. The device without ET host did not work well as OLEDs due to poor electron transport of HT host. The characteristic of OLEDs sets I, II and III are summarized in Table 1, and current-voltage-luminescence curves and luminous loss of device are shown in supplementary information (Supplementary Fig. S1). For all the devices, weight percent of doping concentration is maintained as 10 wt%. As expected, the formation of exciplex improve current efficiency when the mixing ratio of ET to HT is well balanced, as enabling the efficient charge transfer from the exciplex to Ir(TMSBppy)ppy_2_^21,24^. The triplet energy level of exciplex is lower than that of ET or HT host for every device set as shown in Supplementary Table S1 and close to energy level of dopant, hence make energy transfer to dopants efficient. Current efficiency at the mixing ratio of HT to ET molecules, 8:2 dramatically decrease compared to the case of HT to ET 2:8. This result implies that energy transfer from ET host to dopant is more efficient than that from the HT host.

**Table 1:** Summary of performances of studied top emitting OLEDs. (at 10^3^ cd/m2).

Mixing ratio (wt%) of Device series I, II, III	Operating Voltage (V)	Current density (mA/cm^2)^	Current efficiency (cd/A)	Power efficiency (lm/W)	EQE (%)	CIE *x*	CIE *y*	Roll-off (%)	t_95_ (hrs)
PTZP-PCZ:DP-BCZ									
2:8	5.7	18.8	47.8	26.2	11.4	0.283	0.684	0%	165
3:7	5.2	13.2	68.4	41.6	16.5	0.259	0.699	2%	469
4:6	4.7	9.3	96.8	64.4	23.1	0.261	0.699	8%	739
5:5	4.7	8.7	103.0	68.4	24.7	0.252	0.705	5%	365
6:4	4.8	8.6	105.1	69.5	25.0	0.255	0.703	15%	263
7:3	4.7	9.0	100.2	67.4	24.2	0.242	0.710	17%	206
8:2	4.8	9.4	96.2	62.9	23.2	0.242	0.711	21%	182
10:0	5.3	11.4	79.1	46.5	18.7	0.252	0.706	34%	98
PTZP-BCZ:BPP-BCZ									
2:8	5.7	20.0	45.0	24.6	11.0	0.271	0.693	25%	80
3:7	5.0	12.4	72.4	45.1	17.4	0.262	0.700	8%	281
4:6	4.7	10.2	88.2	59.3	21.5	0.239	0.712	13%	453
5:5	4.6	9.7	93.1	64.1	22.8	0.232	0.717	14%	440
6:4	4.8	10.3	87.5	57.5	22.0	0.216	0.723	18%	284
7:3	4.7	10.5	86.1	57.7	21.7	0.211	0.725	19%	145
8:2	4.8	10.8	83.0	54.1	21.2	0.208	0.726	20%	80
10:0	5.2	10.6	70.8	42.4	17.7	0.209	0.727	20%	41
BTPP-PCZ:BPP-BCZ									
2:8	5.9	21.8	45.9	24.4	10.9	0.310	0.664	2%	92
3:7	5.3	15.1	66.1	39.3	15.7	0.293	0.677	6%	291
4:6	5.0	12.4	80.9	51.2	19.3	0.281	0.685	12%	437
5:5	4.8	11.3	88.7	57.9	21.1	0.279	0.687	14%	531
6:4	4.7	11.6	86.6	57.6	21.0	0.259	0.699	17%	473
7:3	4.8	12.1	82.8	54.8	20.0	0.260	0.698	22%	353
8:2	4.8	13.0	76.8	50.1	18.6	0.256	0.701	28%	273
10:0	5.1	15.3	65.3	40.5	15.8	0.253	0.703	41%	90

Device set I including PTZP-PCZ molecules display higher current efficiency irrespective of weight ratio to HT host, compared to other ET hosts. More interestingly, note that device set I exhibits the highest current efficiency at the weight ratio of 6:4, HT to ET, while other device sets which shows the best current efficiency at 5:5. In order to reveal the origin of optimized ratio of ET to HT, we compare charge transport properties of HT and ET host when controlling the mixing ratio. Hole only devices (HODs) and electron only devices (EODs) were fabricated to assess single carrier transport in each ET and HT host, respectively (Fig. 3c). The device structures of HOD and EOD are shown in Supplementary Fig. S2. At high HT host concentration, 0.8 and 0.9, the current density of devices were also not obtained because of device failure. Carrier transport of ET host are far superior to that of HT host based on the current density which is in a good agreement with previous result (Fig. 3a) that current efficiency in ET host only device is higher than that in the device with mixing ration of 2:8 (ET:HT). As increasing mixing ratio of exotic hosts, HT host for EOD, ET host for HOD, current density decreased due to reduction of conduction path of each carrier. The degree of current density decreases in EOD, however, is much higher than that of HOD as increasing the amount of mixed exotic host because ET host with a HOMO level difference of less than 0.2 eV can also be conduction paths for hole in HOD while HT host acts as blocking site for electron due to its shallow LUMO level in EOD. It is notable that EOD exhibit higher current density in an order of PTZP-PCZ > PTZP-BCZ > BTZP-PCZ, corresponding to charge transport property of each ET host, while each HT host shows similar current density at an operating voltage of 5 V. This difference in current density can be attributed to two main factors: charge transport property of ET host and injection barrier at the interface of each layer. Although electron injection barrier of PTZP-BCZ is the lowest among three types of ET host confirmed by slight red shift in PL spectra of the exciplex, the electron current density of PTZP-BCZ is in the middle of three ET host. Therefore, the order of current density among EOD devices is speculated to directly indicate the electron transporting property of each ET host, which is in a good agreement with our aforementioned ET host properties. An ET host with better carrier transport, PTZP-PCZ is expected to achieve charge balance with HT host at a ratio of 4:6 as controlling the amount of ET host molecule due to its superior to HT layer in charge transport, hence exhibit higher efficiency in an exciplex system as shown in Fig. 3. These results suggest that charge balance with a controlled mixing ratio should be considered as an important factor in the exciplex system to optimize current efficiency based on the transport property of each host.

**Figure 4:**
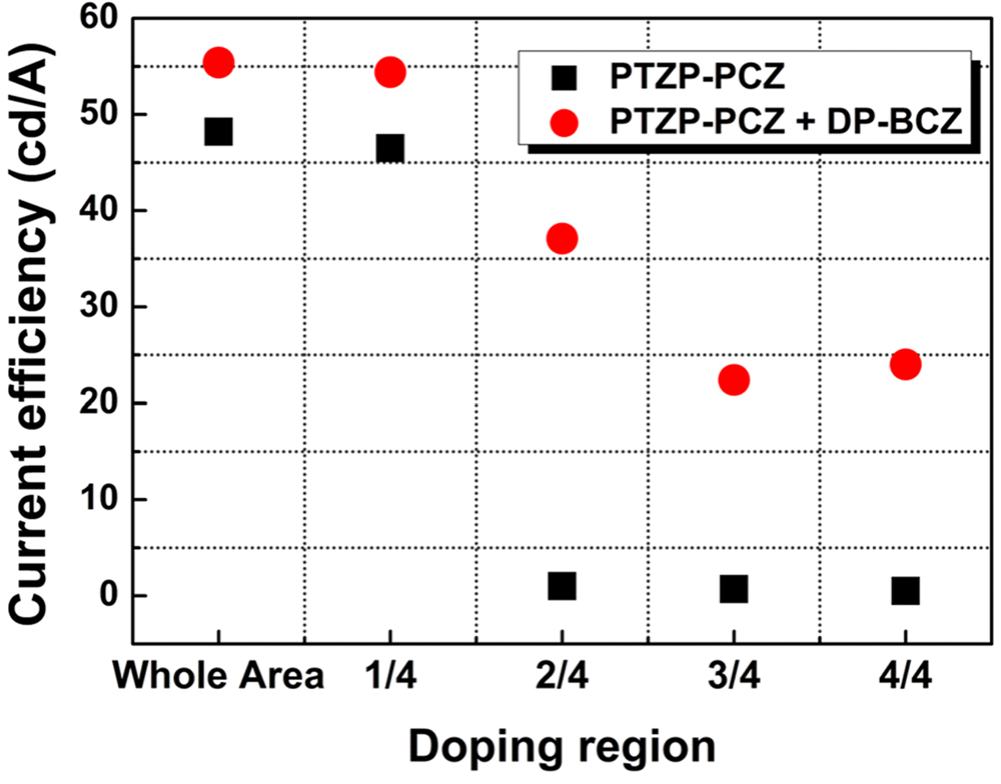
Current efficiencies of fully-doped and partially-doped devices indicating the distribution of recombination zone of single host (PTZP-PCZ, ET host only) and co-host (PTZP-PCZ:DP-BCZ, ET + HT host, 1:1 mixing ratio). Doping regions in EMLs are divided into 4 sections and EMLs are doped separately with stepwise doping profile from HTL to ETL side.

To further investigate the role of HT host on the current efficiency in addition to better energy transfer in exciplex system, we compared two devices without and with HT host with a mixing ratio of 1:1, respectively, controlling the position of dopant in the EML in the combination of device set I. Since the optical resonance effect of top emission OLED can affect the spatial distribution of recombination zone, bottom emission structure was employed. In case of the bottom emission OLEDs, we used transparent ITO anode (150 nm thickness) and Al cathode (60 nm thickness), and layer structures were the same as top emission OLEDs. As described in Fig. 4, the current efficiency in a co-host device is 10% higher than that in ET single-host device when dopants are located in whole EML as well as near the HTL (1/4 doping in Fig. 4) thanks to the formation of exciplex. As the doping position is moved toward ETL in EML, the current efficiency decreases for both single-host and exciplex device. This result clearly indicates the recombination zone is favorable to form at the interface between HTL and EML. Note that the current efficiency in the ET single-host dramatically decreases to the level of less than 1 cd/A, irrespective of the dopant position as dopant position move away from the interface of HTL more than 1/4, while the device with co-host exhibits the efficiency of 37 cd/A, 22 cd/A and 24 cd/A at 2/4, 3/4, and 4/4 dopant position, corresponding to 68%, 41%, and 44% compared to fully doped device, respectively. These results suggest that recombination zone was raised at the HTL/EML interface in ET single host, and shifted toward center of EML from HTL side and broadened to whole EML in HT-ET co-host. Furthermore, this result is also attributed to the property of charge transport in the emission layer. Due to the lack of HT host in the emission layer, hole cannot transport facilely in the emission layer and go through annihilation at the interface, resulting in low efficiency of ET single host device. Although HOMO level of dopant is lower than HT molecule, dopant is expected to act as a trap state in our exciplex system, rather than transport path for hole, resulting in dramatic decrease of current efficiency in the device without HL host. Introduction of HT host in the EML therefore, enhance the hole transport to form exciton in EML as well as shift the emission zone to the center of EML and broaden the recombination zone. Thus, the exciton quenching by triplet-triplet annihilation (TTA) in the EML and triplet-polaron annihilation (TPA) in HTL/EML interface can be reduced and hence the current efficiency can be improved.

Maximum lifetime of device set I, II, III was obtained in a specific molecular mixing ratio, similar manner to the device efficiency, respectively. When either HT or ET is dominant in weight ratio such as 9:1 and 8:2, the lifetime of device dramatically decreased. On the other hands, Lifetime (t_95_) of device set I, II, III was increased from 98 hrs, 41 hrs, 90 hrs to 739 hrs 453 hrs, 531 hrs as DP-BCZ, BPP-BCZ, BPP-BCZ mixing ratio changed from 0 to 6, 6, 5, respectively as shown in Fig. 3a and Table 1. Co-host devices exhibit more than 6 times lifetime compared to ET single host. The dramatic increase of the lifetime can be attributed to the charge balance optimization and uniform exciplex formation in the co-host systems. In the device with an unbalanced mixing ratio of HT/ET host such as 2:8, 3:7 or vice versa, aggregation of the ET or HT host occur in the emission layer, thus reduce the lifetime. Maximum lifetime of device was achieved at the mixing ratio of HT/ET host, 5:5 for device set II and 6:4 for device set I and III, respectively. Since PL spectra does not show extra shoulder peak corresponding to the aggregation of host molecule in the range of HT/ET mixing ratio, 6:4 to 4:6 (Fig. 2a), the achieved maximum lifetime is mainly ascribed to uniform formation of the exciplex irrespective of the combination of HT/ET in devices. As previously explained, the exciton distribution in EML moved toward the center of EML from HTL side and was broadened when HT/ET host mixing ratio was optimized in co-host OLEDs. Therefore, the probability of exciton quenching by TTA and TPA can be reduced, which is well known to give rise to degradation at the interface of HTL/ETL, thus enhance the lifetime of a device. Exciplex normally has a lower energy gap than that of each monomer without perturbing *T*_1_, which decreases the exciton binding energy of the triplet state^34^. Due to the reduced binding energy of *T*_1_ in an exciplex, the surplus energy for exciton formation is decreased and the thermal energy released by non-radiative decay of the exciton will also be reduced, which leads to the increased lifetime of OLED.

Although significant improvement of device lifetime has been achieved by optimizing the mixing ratio of co-host system, the degree of the enhancement in device lifetime showed distinct behavior at a different composition of co-host. For example, when BPP-BCZ was mixed to PTZP-BCZ and BTPP-PCZ, lifetime increased ca. 10 times in BPP-BCZ:PTZP-BCZ, 6 times in BPP-BCZ:BTPP-PCZ co-hosts, respectively, compared to PTZP-BCZ, BTPP-PCZ single hosts. To further investigate this phenomenon, the electronic structures of dimer packing configurations of HT, ET molecules and its exciplex were simulated to elucidate the excited states which are perturbed by HT-ET host interaction due to the exciplex formation. The electronic configurations of BTPP-PCZ:BPP-BCZ, PTZP-BCZ:BPP-BCZ, and PTZP-PCZ:DP-BCZ exciplex are shown in Supplementary Fig. S3. In order to reveal the relationship between device lifetime and excited energy states, energy level calculations of 11 combinations of HT, ET dimers and HT-ET complex were also performed as summarized in Supplementary Tables S2 and S3. The formation of exciplex was identified by analyzing the change of *S*_1_ in the HT-ET complex. As a result, we confirmed that the exciplex was formed when the following condition was satisfied in our test set:

2 $${\rm{\min }}({S}_{1,{\rm{HT}}},{S}_{1,{\rm{ET}}})\,-\,{S}_{1,{\rm{HT}} \mbox{-} {\rm{ET\; complex}}}\,\ge \,0.15\,{\rm{eV}}$$

**Figure 5:**
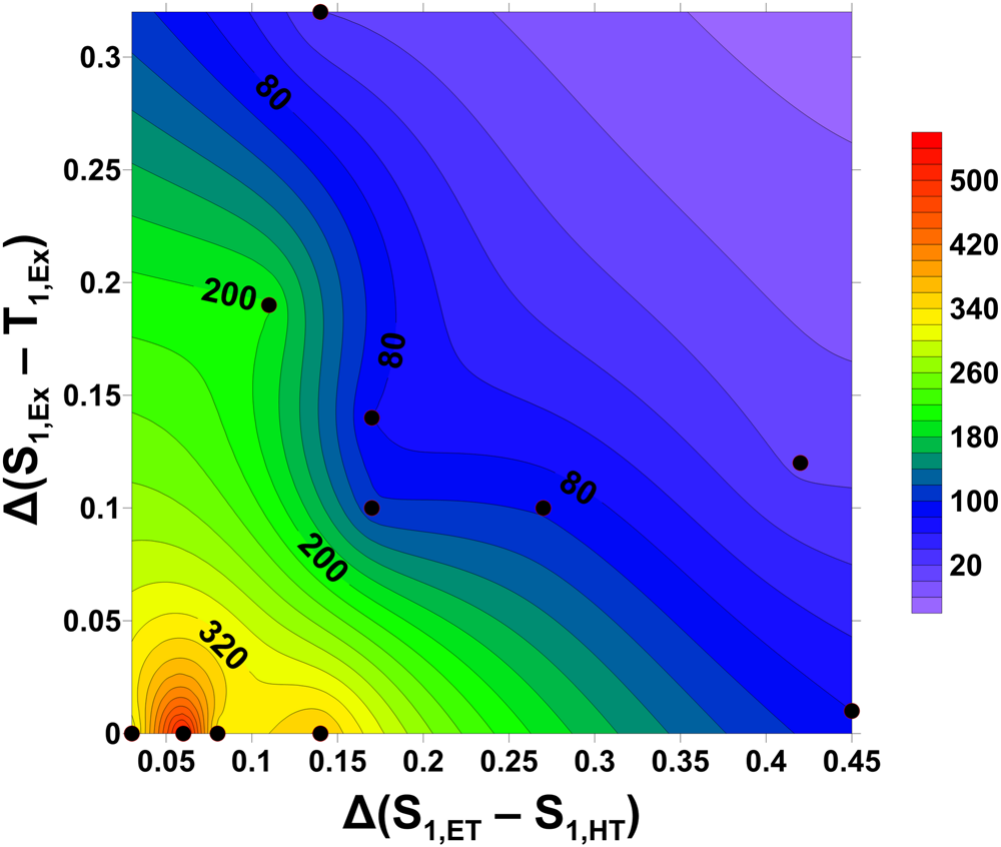
Correlation between relative device lifetime (t_95_) and excitation energy perturbed by exciplex formation

The relation of the device lifetime with a *S*_1_ difference between ET and HT host [Δ(*S*_1,ET_ − *S*_1,HT_)] and *S*_1 −_ *T*_1_ gap in HT-ET complex [Δ(*S*_1,Ex_ − *T*_1,Ex_)] is shown in Fig. 5. We found that there was a close relationship between Δ(*S*_1,ET_ − *S*_1,HT_), Δ(*S*_1,Ex −_ *T*_1,Ex_) and lifetime of devices. As Δ(S_1,ET_ − S_1,HT_), Δ(S_1,Ex_ − T_1,Ex_) decrease, lifetime of devices is dramatically enhanced when Δ(S_1,ET_ − S_1,HT_), Δ(S_1,Ex_ − T_1,Ex_) are less than 0.15 eV. When the difference of *S*_1_ between HT and ET is small, exciton-induced degradation due to the hot molecules of host with high singlet excited energy can be suppressed^35,36^. In addition, as the energy gap between *S*_1_ and *T*_1_ of HT-ET complex is small, the value of the orbital overlap integral (the exchange energy of co-host) is reduced and the fluorescence decay time will be increased^37^. As a result, unnecessary fluorescence decay of the host is minimized and the energy transfer from the host to the dopant can be increased. As the energy transfer becomes efficient in the co-host system, the probability of non-radiative decay causing thermal degradation will be reduced, thus the lifetime of the devices can be enhanced. Furthermore, small ΔE_st_ also has been reported to enhance up-conversion to the singlet states and transfer to the guests, thus reduce exciton quenching and efficiency roll-off, and hence extend the lifetime^38^.

## Conclusions

In conclusion, high efficiency and long lifetime in phosphorescent OLEDs were demonstrated using the exciplex forming mixed host EML system as introducing new types of band-engineered material systems of PTZP-PCZ:DP-BCZ, PTZP-BCZ:BPP-BCZ and BTPP-PCZ:BPP-BCZ. Regarding the color pixel designs of commercialized OLED panels, a hole injection/transport layer (HIL/HTL) and electron injection/transport layer (EIL/ETL) are used as a common layer for each red, green, and blue pixels (EML) to reduce the number of color patterning process, thus the number of unit processes and cost of production. When the HIL/HTL and EIL/ETL are fixed as common layers for EMLs, the charge balance can be controlled only by the transport/injection properties of EML host materials for each color. Normally, it is difficult to control charge injection and transport properties of hole and electron in bipolar host materials since the electron and hole injection/transport properties cannot be controlled individually in one material. With exciplex-forming co-host systems, we demonstrate a pathway to control the charge balance optimization by controlling HT/ET mixing ratio in EML. Our investigation propose that mixing ratio of HT/ET host should be adjusted depending on relative charge transport property of ET/HT host to achieve charge balance in the emission layer for highly efficient device employing exciplex system. Furthermore, new parameters for describing exciton stability due to the exciplex formation were also suggested. Our comprehensive study shows that energy gap between singlet and triplet in exciplex system as well as that in each host should be considered to enhance the lifetime of OLEDs. Our approach can pave the way for developing peculiar co-host materials for highly efficient and lifetime-enhanced phosphorescent organic light emitting diode.

## Experimental

Top emitting OLEDs are fabricated on patterned Ag/ITO substrates. HILs (2 wt% NDP9, NDP series, Novaled AG, Dresden, N-([1,1′-biphenyl]-4-yl)-9,9-dimethyl-N-(4-(9-phenyl-9H-carbazol-3-yl)phenyl)-9H-fluoren-2-amine(BCFA))/HTLs(BCFA)/EMLs/ETLs(1,3,5-tris(N-phenylbenzimidazole-2yl)benzene (TPBi) mixed with lithium quinolate(Liq, 1:1 wt.% ratio))/EILs(Liq)/Mg:Ag(10:1 ratio)/capping layers are thermally evaporated under pressure of 1 × 10–7 torr sequentially. For EML, 5-(3–4,6-diphenyl-1,3,5-triazin-2-yl)phenyl-3,9-diphenyl-9H-carbazole (PTZP-PCZ), 2-(3-(6,9-diphenyl-9H-carbazol-4-yl)phenyl)-4-phenylbenzo[4,5]thieno[3,2-d]pyrimidine (BTPP-PCZ), and 5-(3–4,6-diphenyl-1,3,5-triazin-2-yl)phenyl)-9-phenyl-9H-3,9′-bicarbazole (PTZP-BCZ) are used as ET hosts and 9,9′-dipenyl-9H, 9’H-3,3’-bicarbazole (DP-BCZ) and 9-([1,1′-biphenyl]-4-yl)-9′-phenyl-9H,9′H-3,3′-bicarbazole (BPP-BCZ) are used as HT host. PTZP-PCZ is co-deposited with BP-BCZ, and BTPP-PCZ and PTZP-BCZ are co-deposited with BPP-BCZ. For emitter, {2-([1,1′-biphenyl]-3-yl)-5-(trimethylsilyl)pyridinato-C2,N}-bis[2-phenylpyridinato-C2,N]iridium(III), Ir(TMSBppy)ppy2 is doped with the concentration of 10 wt%. The thickness of each layers are shown in Fig. 1. To describe the nature of the exciplex formation, the monomer structure of host materials and their dimer configurations including an exciplex or a non-exciplex geometry were calculated. The optimized configuration was obtained by selecting the pair with minimum interaction energy among arbitrarily generated configurations by using the Blends module with DREIDING force-field in Materials Studio package. During the calculations, energetically favorable dimer configurations were predominantly obtained by using a sampling technique based on the excluded-volume constraints method^39^ and the molecular Silverware algorithm^40^. Subsequently, based on the optimal dimer configurations, their electronic structures were calculated by using B3LYP functionals with 6–31 G** basis set in the density functional theory (DFT) formalism in Gaussina09 package. The restricted time-dependent B3LYP calculations were used to obtain the excitation energies of exciplexes. The lifetimes of the OLEDs were measured by the Mcscience OLED Lifetime Test System (Polaronix^®^ M6000PMX, Korea) for continuous operation at a constant current and room temperature.

## Electronic supplementary material

Supplementary information
