# Supplementary material for: The Role of Charge Balance and Excited State Levels on Device Performance of Exciplex-based Phosphorescent Organic Light Emitting Diodes
Source: Sci Rep. 2017 Sep 20;7:11995. doi: 10.1038/s41598-017-12059-2 (PMC5607313; doi:10.1038/s41598-017-12059-2)
Supplement: Supplementary file 1 — Supplementary information [file 41598_2017_12059_MOESM1_ESM.doc]

**The Role of Charge Balance and Excited State Levels on Device Performance of Exciplex-based Phosphorescent Organic Light Emitting Diodes**

Sangyeob Lee^1†^, Hyun Koo^1†^, Ohyun Kwon^1^, Young Jae Park^1^, Hyeonho Choi^1^, Kwan Lee^2^, Byungmin Ahn^2*^, and Young Min Park ^1,3*^

^1^Samsung Advanced Institute of Technology, Samsung Electronics Co., Ltd, 130 Samsung-ro, Yeongtong-gu, Suwon, Gyeonggi, 16678, Korea

^2^Department of Materials Science and Engineering and Department of Energy Systems Research, Ajou University, 206 Worldcup-ro, Yeongtong-gu, Suwon, Gyeonggi, 16499, Korea

^3^Surface Technology Group, Korea Institute of Industrial Technology (KITECH), Incheon 21999 Republic of Korea

**Supplementary Information**

**Supplementary Figure S1.** (a) *J*-*V* and *L*-*V*, (b) Current efficiency vs. *J*, (c) Normalized luminance loss of device set PTZP-PCZ:DP-BCZ, PTZP-BCZ:BPP-BCZ and BTPP-PCZ:BPP-BCZ as function of HT-ET host mixing ratio. For simplicity, device properties with mixing ratio of 7:3, 5:5, 3:7 (HT host:ET host) are selectively shown (8:2, 6:4, 4:6, 2:8 of HT host:ET host mixing ratio are not presented).

**
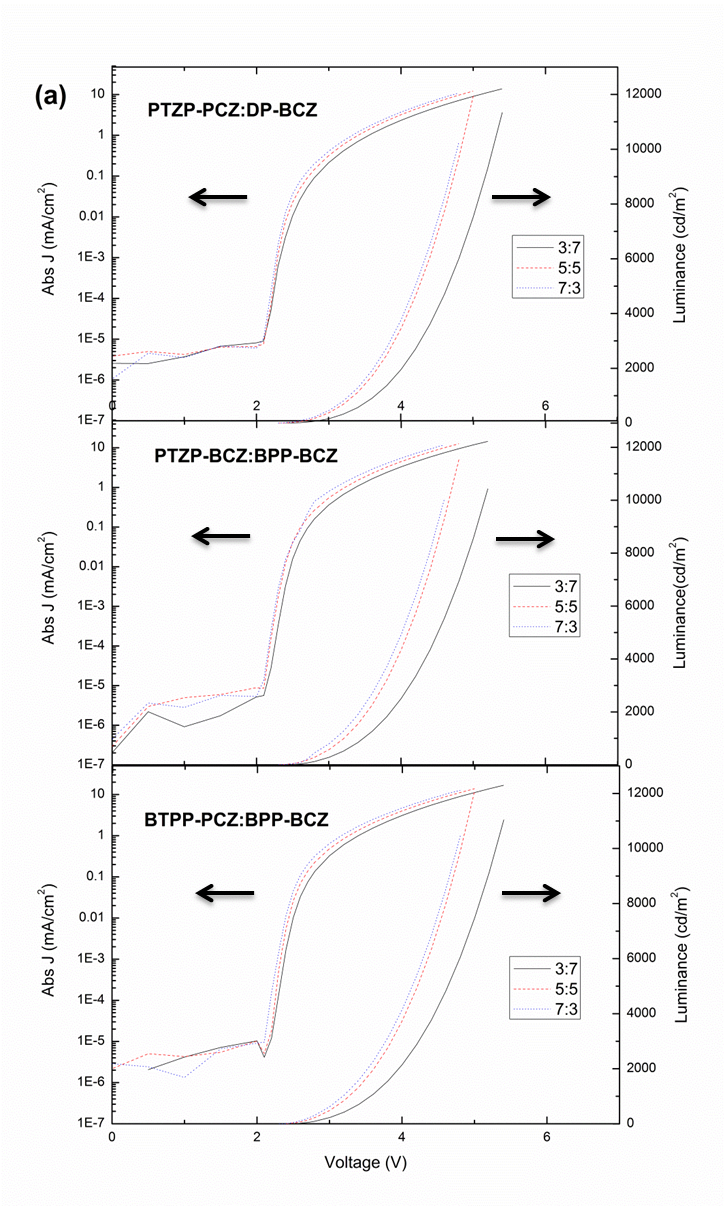
**

**
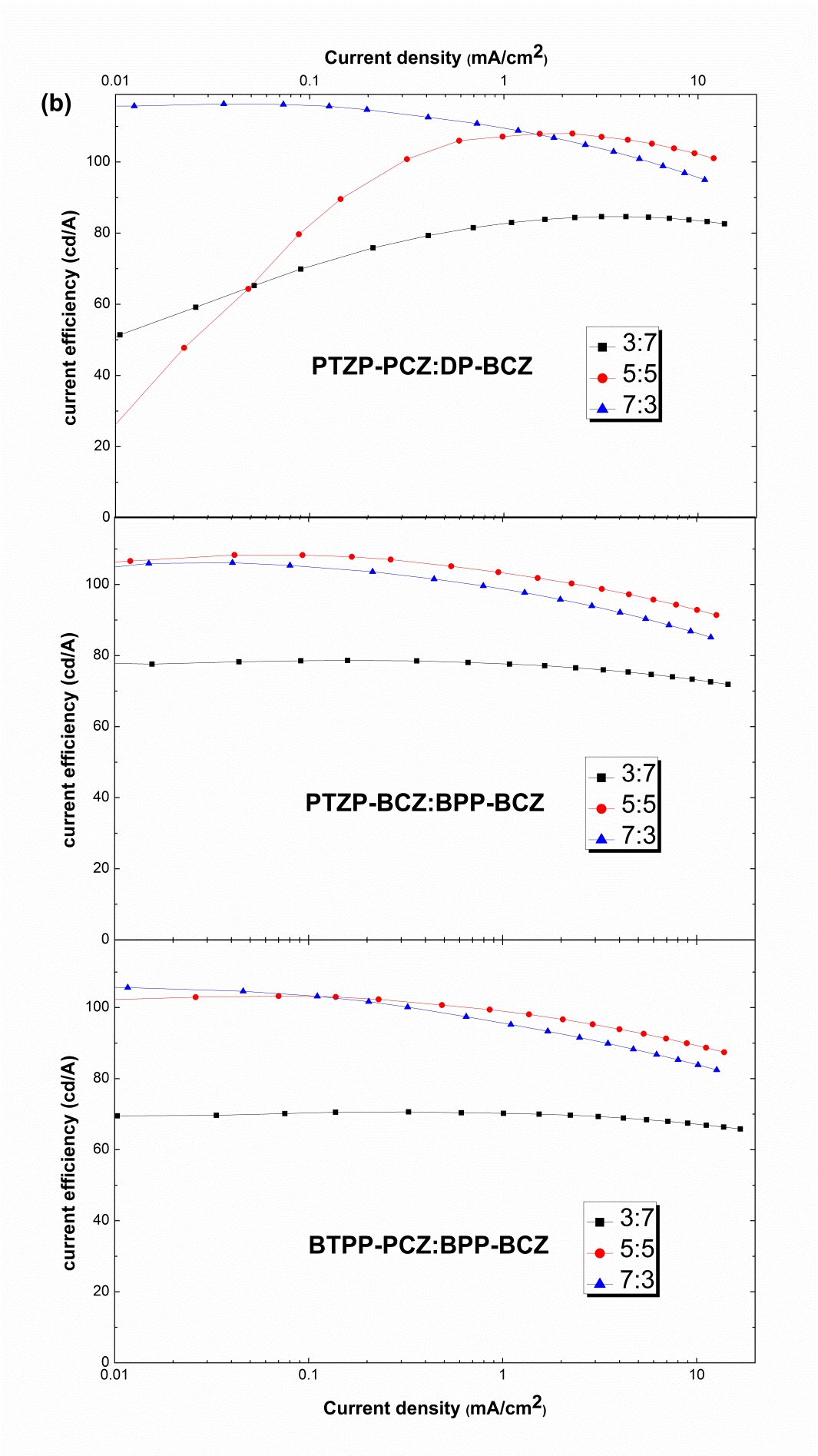
**

**
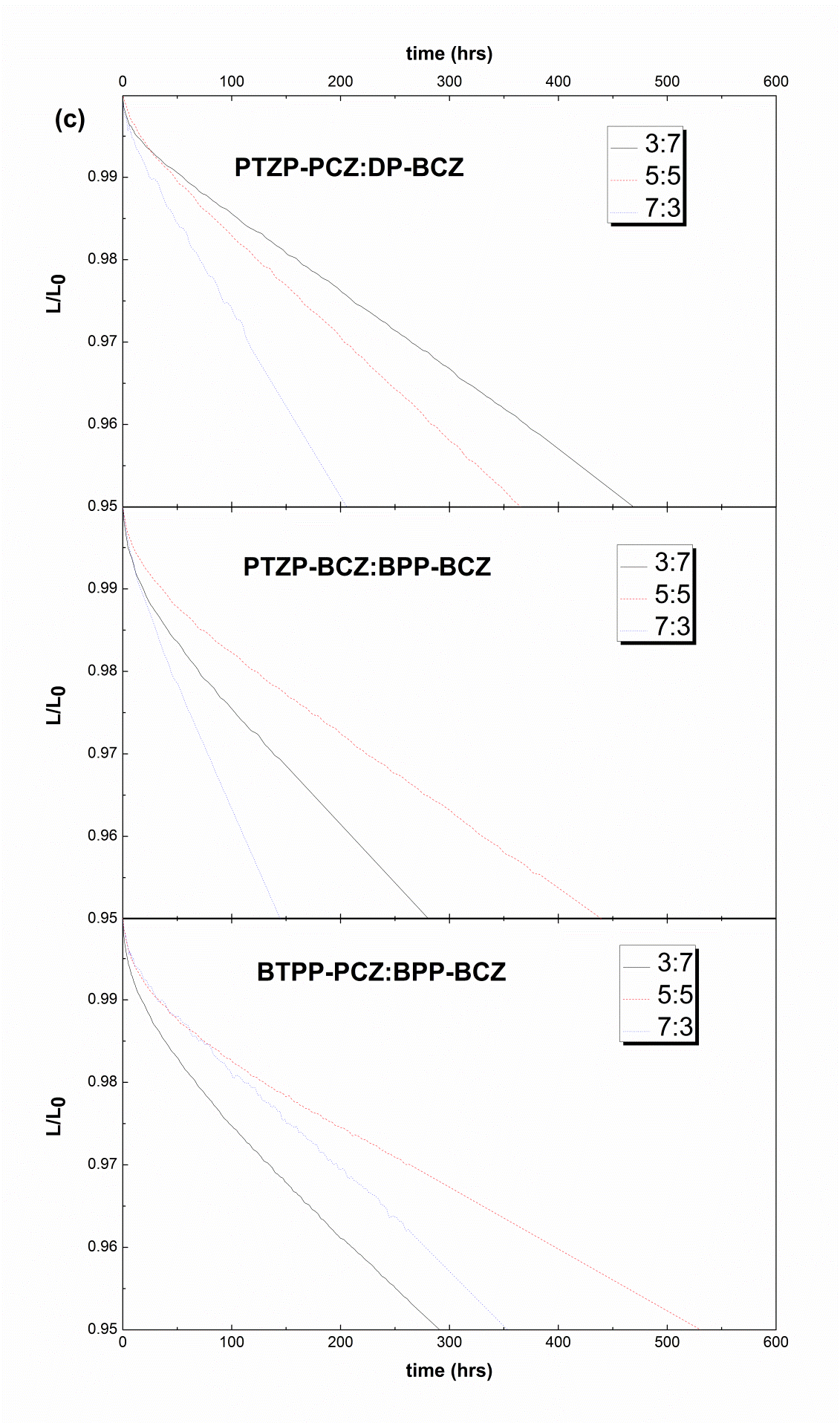
**

**Supplementary Figure S2.** Device structure of (a) HOD and (b) EOD.


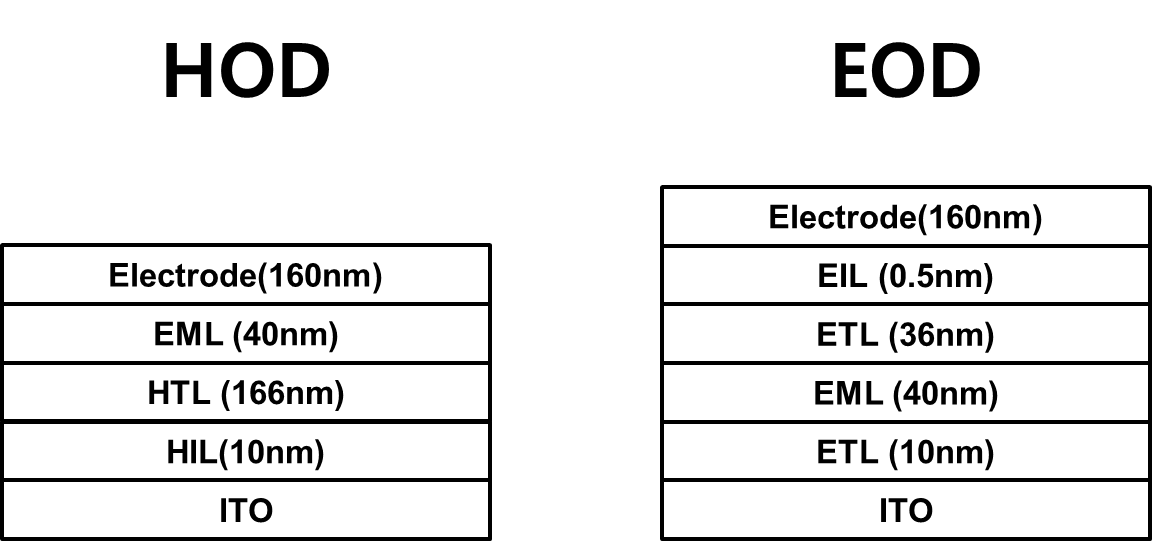


**Supplementary Figure S3.** Dimer packing configuration of (a) BTPP-PCZ:BPP-BCZ, (b) PTZP-BCZ:BPP-BCZ, (c) PTZP-PCZ:DP-BCZ and its frontier molecular orbitals (HOMO (left) and LUMO(right)).


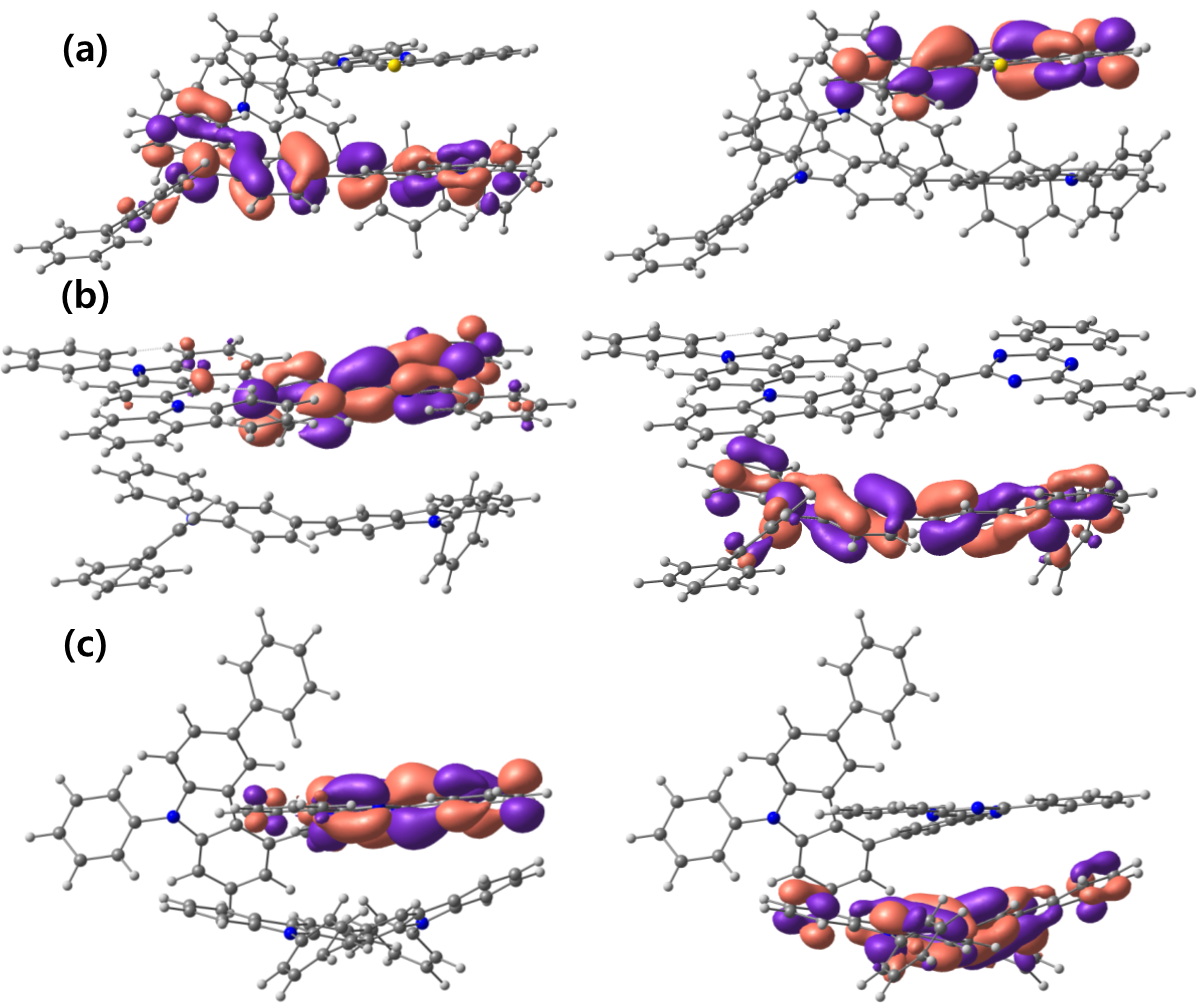


**Supplementary Figure S4.** Molecular structures of the organic compound used in Table S1


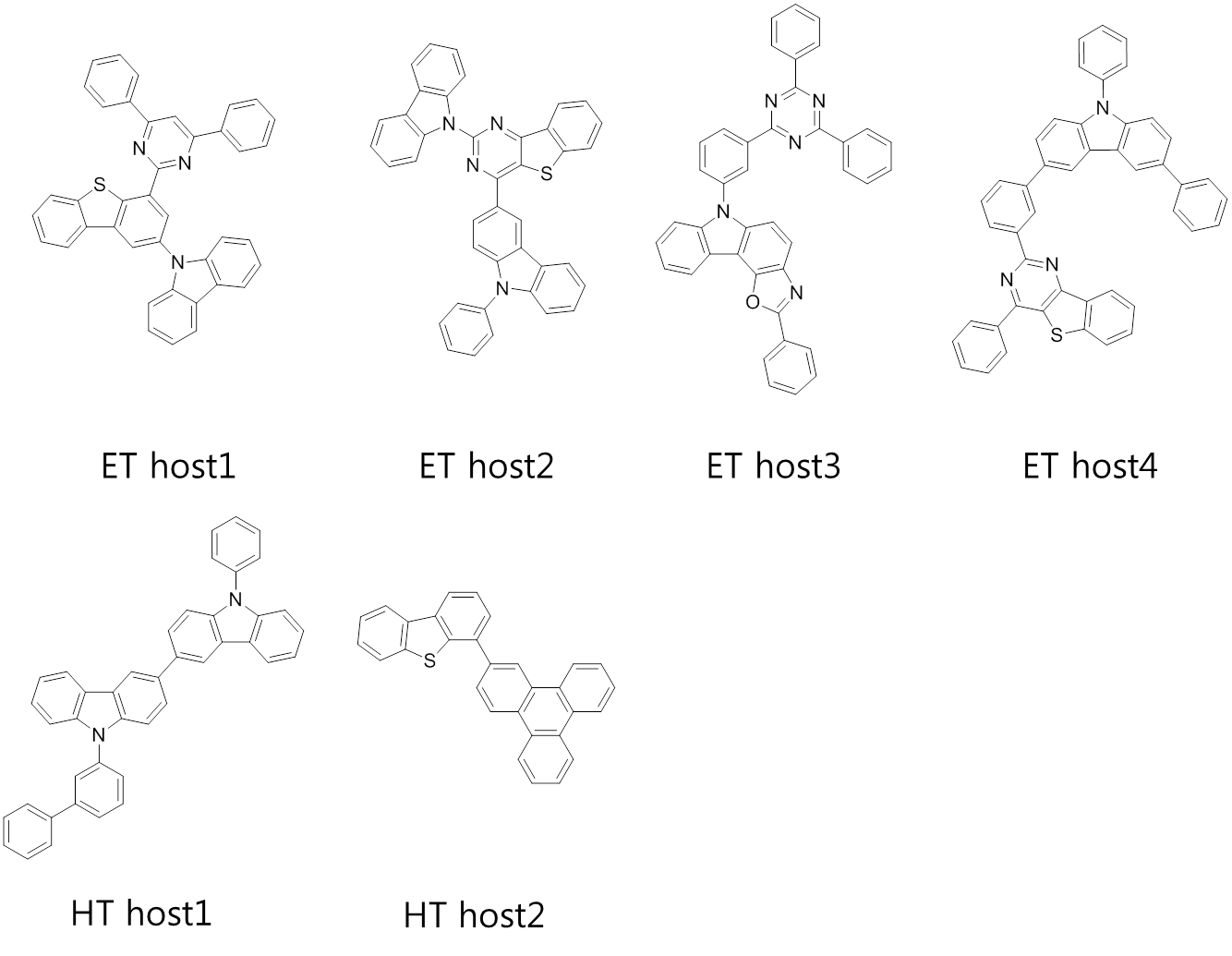


ET host1: 9-(4-(4,6-diphenylpyrimidin-2-yl)dibenzo[b,d]thiophen-2-yl)-9H-carbazole

ET host2: 2-(9H-carbazol-9-yl)-4-(9-phenyl-9H-carbazol-3-yl)benzo[4,5]thieno[3,2-d]pyrimidine

ET host3: 6-(3-(4,6-diphenyl-1,3,5-triazin-2-yl)phenyl)-2-phenyl-6H-oxazolo[4,5-c]carbazole

ET host4: 2-(3-(6,9-diphenyl-9H-carbazol-3-yl)phenyl)-4-phenylbenzo[4,5]thieno[3,2-d]pyrimidine

HT host1: 9-([1,1'-biphenyl]-3-yl)-9'-phenyl-9H,9'H-3,3'-bicarbazole

HT host2: 4-(triphenylen-2-yl)dibenzo[b,d]thiophene

**Supplementary Table S1.** Measured and calculated Values of HOMO, LUMO, S1, T1 of ET/HT host materials. All units in eV.

|  | **Experiment** | | | | **Calculation** | | | |
| --- | --- | --- | --- | --- | --- | --- | --- | --- |
|  | **HOMO** | **LUMO** | **S1** | **T1** | **HOMO** | **LUMO** | **S1** | **T1** |
| **PTZP-PCZ** | -5.95 | -2.43 | 2.81 | 2.65 | -5.195 | -1.82 | 2.958 | 2.799 |
| **PTZP-BCZ** | -5.78 | -2.4 | 2.7 | 2.65 | -5.054 | -1.809 | 2.805 | 2.799 |
| **BTPP-PCZ** | -5.86 | -2.41 | 2.78 | 2.74 | -5.129 | -1.819 | 2.931 | 2.864 |
| **DP-BCZ** | -5.5 | -1.24 | 3.14 | 2.95 | -4.973 | -0.69 | 3.253 | 3.028 |
| **BPP-BCZ** | -5.51 | -1.31 | 3.1 | 2.88 | -4.977 | -0.748 | 3.215 | 2.994 |

**Supplementary Table S2.** Calculated values of HOMO, LUMO of HTL, ETL, and HT/ET host materials. All units in eV.

| **Material** | **HOMO** | **LUMO** |
| --- | --- | --- |
| HTL | -4.692 | -0.903 |
| ETL | -5.800 | -1.940 |
| PTZP-PCZ | -5.195 | -1.82 |
| PTZP-BCZ | -5.054 | -1.809 |
| BTPP-PCZ | -5.129 | -1.819 |
| DP-BCZ | -4.973 | -0.690 |
| BPP-BCZ | -4.977 | -0.748 |
| ET host 1 | -5.233 | -1.829 |
| ET host 2 | -5.316 | -1.776 |
| ET host 3 | -5.262 | -2.023 |
| ET host 4 | -5.173 | -1.775 |
| HT host 1 | -4.972 | -0.95 |
| HT host 2 | -5.624 | -1.24 |

**Supplementary Table S3.** Calculated values of *S*_1_, *T*_1_ of ET host dimer, HT host dimer, and exciplex (HT-ET complex). All units in eV expect for *t*_95_ (hrs).

| **ET host** | **HT host** | **ET host dimer** | | **HT host dimer** | | **HT/ET complex** | | **Δ(S_1,ET_ – S_1,HT_)** | **Δ(S_1,Ex_ – T_1,Ex_)** | ***t*_95_**  **_(hrs)_** |
| --- | --- | --- | --- | --- | --- | --- | --- | --- | --- | --- |
|  |  | **S_1_** | **T_1_** | **S_1_** | **T_1_** | **S_1_** | **T_1_** |  |  |  |
| **PTZP-PCZ** | **DP-BCZ** | 3.01 | 2.92 | 3.15 | 3.02 | 2.71 | 2.71 | 0.14 | 0.00 | 365 |
| **PTZP-BCZ** | **BPP-BCZ** | 2.98 | 2.97 | 3.04 | 3.00 | 2.68 | 2.68 | 0.06 | 0.00 | 440 |
| **BTPP-PCZ** | **BPP-BCZ** | 2.98 | 2.84 | 3.04 | 3.00 | 2.65 | 2.65 | 0.06 | 0.00 | 531 |
| **ET host 1** | **DP-BCZ** | 2.88 | 2.70 | 3.15 | 3.02 | 2.83 | 2.73 | 0.27 | 0.10 | 97 |
| **ET host 2** | **DP-BCZ** | 2.98 | 2.66 | 3.15 | 3.02 | 2.77 | 2.63 | 0.17 | 0.14 | 80 |
| **ET host 3** | **DP-BCZ** | 2.70 | 2.61 | 3.15 | 3.02 | 2.55 | 2.54 | 0.45 | 0.01 | 80 |
| **ET host 4** | **BPP-BCZ** | 2.96 | 2.84 | 3.04 | 3.00 | 2.75 | 2.75 | 0.08 | 0.00 | 320 |
| **PTZP-PCZ** | **BPP-BCZ** | 3.01 | 2.92 | 3.04 | 3.00 | 2.66 | 2.66 | 0.03 | 0.00 | 300 |
| **ET host 2** | **HT host 1** | 2.98 | 2.66 | 3.15 | 3.02 | 2.74 | 2.64 | 0.17 | 0.10 | 106 |
| **PTZP-PCZ** | **HT host 1** | 3.01 | 2.92 | 3.15 | 3.02 | 2.66 | 2.66 | 0.14 | 0.00 | 262 |
| **BTPP-PCZ** | **HT host 2** | 2.98 | 2.66 | 3.12 | 2.73 | 2.96 | 2.64 | 0.14 | 0.32 | 20 |
| **ET host 3** | **HT host 2** | 2.70 | 2.61 | 3.12 | 2.73 | 2.77 | 2.65 | 0.42 | 0.12 | 15 |
| **PTZP-PCZ** | **HT host 2** | 3.01 | 2.92 | 3.12 | 2.73 | 2.93 | 2.74 | 0.11 | 0.19 | 202 |

**Synthetic Route and Characterization of Materials**^1,2^

**Synthesis of 5-(3-4,6-diphenyl-1,3,5-triazin-2-yl)phenyl-3,9-diphenyl-9H-carbazole**

**PTZP-PCZ** was synthesized as following; 17.5 g (42 mmol) of 5-bromo-3,9-diphenyl-9H-carbazole, 20.0 g (46 mmol) of 2,4-diphenyl-6-(3-(4,4,5,5-tetramethyl-1,3,2-dioxaborolan-2-yl)phenyl)-1,3,5-triazine, 14 g (104 mmol) of K_2_CO_3_, and 2.4 g (2.1 mmol) of Pd(PPh_3_)_4_ were added to 80 mL of toluene and stirred at about 120℃ for about 48 hours. After completion of the reaction, water was added to the reaction product and stirred, followed by filtration to obtain a dark gray solid. This solid was dissolved in hot toluene, and then filtered. The resulting toluene solution was collected, and methanol was added thereto to obtain a precipitate. This precipitate was filtered to obtain a solid, which was then recrystallized with 1-chlorobenzene to obtain Compound **PTZP-PCZ** in white powder (18.0 g, Yield: 70 %). Resulting material was identified using ^1^H-nuclear magnetic resonance (NMR) and liquid chromatography–mass spectrometry (LC/MS). ^1^H NMR (CDCl_3_, 300MHz): 9.17 (s, 1H), 8.92 (d, 1H), 8.77 (dd, 4H), 7.95~7.68 (m, 7H), 7.66~7.40 (m, 14H), 7.27~7.19 (m, 3H). LC/MS, calculated: C_45_H_30_N_4_ = 626.75, found: m/z = 626.2 (M+, 100 %)

**Synthesis of 5-(3-4,6-diphenyl-1,3,5-triazin-2-yl)phenyl)-9-phenyl-9H-3,9'-bicarbazole**

**PTZP-BCZ** (white solid, Yield: 68 %) was synthesized in the same manner as in the synthesis of PTZP-PCZ via recrystallization with n-methyl-2-pyrrolidone (NMP), except that 5-bromo-9-phenyl-9H-3,9'-bicarbazole, instead of 5-bromo-3,9-diphenyl-9H-carbazole, was used. **PTZP-BCZ** was identified using LC/MS. LC/MS, calcd. : C_51_H_33_N_5_ = 715.84, found: m/z = 715.2 (M+, 100 %)

**Synthesis of 2-(3-(6,9-diphenyl-9H-carbazol-4-yl)phenyl)-4-phenylbenzo[4,5]thieno[3,2-d]pyrimidine**

**BTPP-PCZ** (yellow solid, Yield: 60 %) was synthesized in the same manner as in the synthesis of PTZP-PCZ via recrystallization with n-methyl-2-pyrrolidone (NMP), except that 4-phenyl-2-(3-(4,4,5,5-tetramethyl-1,3,2-dioxaborolan-2-yl)phenyl)benzo[4,5]thieno[3,2-d]pyrimidine, instead of 2,4-diphenyl-6-(3-(4,4,5,5-tetramethyl-1,3,2-dioxaborolan-2-yl)phenyl)-1,3,5-triazine, was used. **BTPP-PCZ** was identified using LC/MS. LC/MS, calcd. : C_46_H_29_N_3_S = 655.21, found: m/z = 655.2 (M+, 100 %)

**Reference**

1 Lee, B., Kim, S., Lee, S., Kwon, O. & Kim, Y. Carbazole-based compound and organic light-emitting device including the same. U.S. patent US20150228908 A1.

2 Lee, S. et al. Organic light-emitting device. U.S. patent US 20160072078 A1.
